# Supplementary material for: Wheat Space Odyssey: “From Seed to Seed”. Kernel Morphology
Source: Life (Basel). 2019 Oct 25;9(4):81. doi: 10.3390/life9040081 (PMC6958380; doi:10.3390/life9040081)

Supplement File 1

1. Cosmonaut S. Volkov near “Lada” space greenhouse aboard the ISS. Growing wheat plants are observed behind a window.

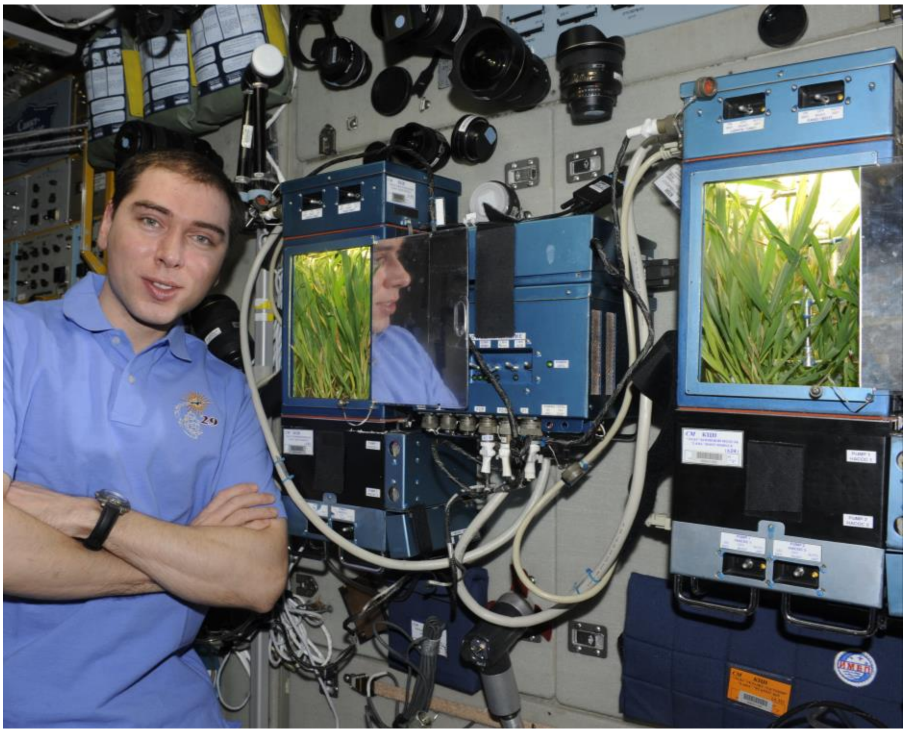

2. Space greenhouse “Lada” with fully matured ears of Super-Dwarf wheat ready for harvest.

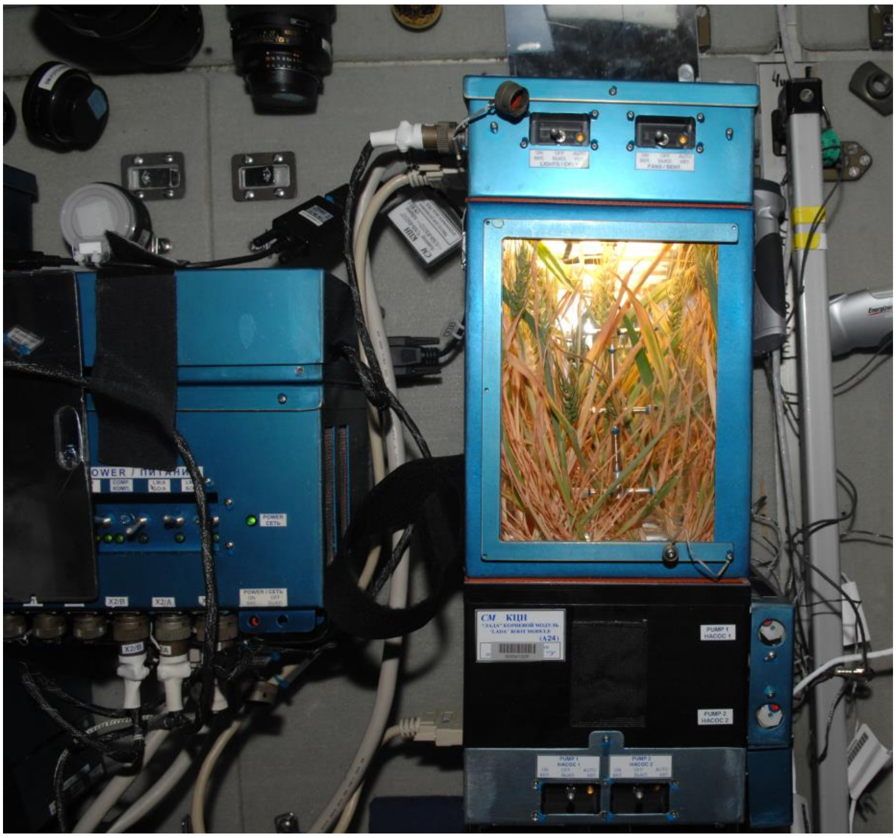

Supplement: Supplementary file 1 [file life-09-00081-s001.pdf]
